# Current and Potential Treatments for Reducing *Campylobacter* Colonization in Animal Hosts and Disease in Humans

**DOI:** 10.3389/fmicb.2017.00487

**Published:** 2017-03-23

**Authors:** Tylor J. Johnson, Janette M. Shank, Jeremiah G. Johnson

**Affiliations:** Department of Microbiology, The University of Tennessee, KnoxvilleTN, USA

**Keywords:** *Campylobacter*, chicken colonization, anti-infectves, food safety, antibiotic resistance

## Abstract

*Campylobacter jejuni* is the leading cause of bacteria-derived gastroenteritis worldwide. In the developed world, *Campylobacter* is usually acquired by consuming under-cooked poultry, while in the developing world it is often obtained through drinking contaminated water. Once consumed, the bacteria adhere to the intestinal epithelium or mucus layer, causing toxin-mediated inhibition of fluid reabsorption from the intestine and invasion-induced inflammation and diarrhea. Traditionally, severe or prolonged cases of campylobacteriosis have been treated with antibiotics; however, overuse of these antibiotics has led to the emergence of antibiotic-resistant strains. As the incidence of antibiotic resistance, emergence of post-infectious diseases, and economic burden associated with *Campylobacter* increases, it is becoming urgent that novel treatments are developed to reduce *Campylobacter* numbers in commercial poultry and campylobacteriosis in humans. The purpose of this review is to provide the current status of present and proposed treatments to combat *Campylobacter* infection in humans and colonization in animal reservoirs. These treatments include anti-*Campylobacter* compounds, probiotics, bacteriophage, vaccines, and anti*-Campylobacter* bacteriocins, all of which may be successful at reducing the incidence of campylobacteriosis in humans and/or colonization loads in poultry. In addition to reviewing treatments, we will also address several proposed targets that may be used in future development of novel anti-*Campylobacter* treatments.

## Introduction

*Campylobacter jejuni* is the leading global cause of gastroenteritis derived from bacteria. The substantial increase of both incidence and prevalence of campylobacteriosis in Europe, Australia, and North America is troubling, and data from Asia, Africa, and the Middle East indicate that campylobacteriosis has become endemic in these areas, especially in young children (Kaakoush et al., 2015). In the United States, treatment of acute disease and post-infectious disorders associated with *Campylobacter* infection, cost approximately $1.7 billion USD annually (Maue et al., 2014). Following ingestion of the bacterium, *Campylobacter* adheres to and invades the epithelial cells lining the gastrointestinal tract, inducing a potent inflammatory response (Backert et al., 2013; Samuelson et al., 2013). This results in moderate to severe diarrhea that may be accompanied by frank blood in the stool, abdominal cramps, and fever. While campylobacteriosis is typically characterized by gastroenteritis, it can also lead to septicemia, post-infectious arthritis, GBS, or Miller Fisher syndrome (Goldstein et al., 2016). Additionally, *Campylobacter* spp. have recently been associated with inflammatory bowel diseases such as Crohn’s disease and ulcerative colitis (Kaakoush et al., 2014a,b).

Illnesses associated with *Campylobacter* are a greater burden in developing countries. While infection in immunocompetent patients in the developed world is usually self-limiting, it has been observed to persist in the gastrointestinal tracts of some patients, particularly young children in the developing world, that leads to stunting (Amour et al., 2016). Similarly, persistent diarrhea and severe bacteremia associated with *Campylobacter* spp. have been observed in HIV/AIDS patients (Coker et al., 2002). As such, morbidity and mortality caused by *Campylobacter* is increased among HIV positive individuals, particularly in the developing world (Tee and Mijch, 1998; Guerry et al., 2012).

In the developed world, *C. jejuni* is a leading cause of food-borne illness primarily due to its ability to asymptomatically colonize agriculturally relevant animals, including chickens (Johnson et al., 2015). In poultry flocks, natural colonization of chicks occurs within 2 – 3 weeks of hatching via horizontal contamination from the environment and birds typically remain colonized for life (Sahin et al., 2003). Since domestic and wild birds are the microorganism’s primary reservoir, they may carry up to 10^9^ CFU *Campylobacter* per gram of cecal contents (Meunier et al., 2016b). The microorganism can then spread from the intestines of poultry to meat during processing.

According to a survey of *Campylobacters* in England and Wales, *C. jejuni* is responsible for approximately 90% of campylobacteriosis cases and *C. coli* are responsible for the remaining 10% (Gillespie et al., 2002). Other *Campylobacter* species can also cause disease, but they are rarely involved (Meunier et al., 2016b). Human infection can occur following ingestion of as few as 500 *Campylobacter* cells; however, the sample size in this particular study was small (*n* = 1) (Robinson, 1981). Another study that determined the infectious dose of *Campylobacter* required to result in diarrhea or fever, found that no clear correlation was observed between dose and the percentage of participants that presented with these symtoms. Similarly, no dose response was observed for colonization as all doses resulted in 100% of humans presenting with positive stool cultures (Black et al., 1988).

Not surprisingly, as chickens serve as a major source of human infections in the developed world, it has been proposed that to decrease the incidence of campylobacteriosis, avian colonization must be combatted (Meunier et al., 2016b). Since it has been predicted that decreasing *Campylobacter* colonization of poultry by 2-log_10_ will reduce human infections by 30-fold, much research has focused on understanding colonization of poultry by *Campylobacter*, since even a small reduction could have an enormously positive impact on human health (Rosenquist et al., 2003). While ingestion of contaminated poultry is the primary mode of infection in developed parts of the world, ingestion of contaminated water is commonly responsible for *Campylobacter* infections in developing parts of the world (Kaakoush et al., 2015).

Since campylobacteriosis is usually self-limiting and treatment with antibiotics typically only decreases the duration of gastrointestinal symptoms by 1.32 days, some groups have advised against antibiotic treatment in uncomplicated campylobacteriosis cases (Ternhag et al., 2007). In severe or prolonged cases that are generally associated with immunocompromised persons or young children, patients are treated with antibiotics from the macrolide (erythromycin) or quinolone (ciprofloxacin) classes (Longenberger et al., 2013; Kovač et al., 2015). Unfortunately, the emergence of antibiotic resistant *Campylobacter* necessitates the development of novel antimicrobials (Kumar et al., 2016). For example, the use of fluoroquinolones in poultry production coincided with the emergence of ciprofloxacin-resistant *Campylobacter* in humans (Moore et al., 2006). As such, the Centers for Disease Control and Prevention reported an increase in ciprofloxacin resistance in *Campylobacter* from 13 to 25% occurred between 1997 and 2011 (Hampton, 2013). More concerning, some European Union Member states reported up to a 91.5% incidence of quinolone resistant *Campylobacter* (EFSA, 2014). Similarly, it was determined that the incidence of ciprofloxacin resistance in *Campylobacter* isolates from raw chicken in South Korea was approximately 92% (Han et al., 2007) and 100% in clinical isolates from children in Thailand (Serichantalergs et al., 2007). Because of this prevalence, international travel-associated infections in the United States are often caused by quinolone-resistant *Campylobacter* isolates, exhibiting resistant rates of 60%; this is compared to the 13% of non-travel related cases (Ricotta et al., 2014). These data show that antibiotic resistant *Campylobacter* is a global issue that has far-reaching effects on human health.

Since the incidence of antibiotic resistance in *Campylobacter* is increasing, the severity of post-infectious disorders is becoming better understood, and the economic burden associated with campylobacteriosis in humans is substantial, it is essential that novel interventions be developed to reduce the incidence of *Campylobacter* colonization in commercial poultry and reduce the number of campylobacteriosis cases in humans. Thus, the purpose of this review is to provide the status of several current and proposed treatments to combat *Campylobacter* infection in humans either directly or through reducing colonization of poultry. We will also address potential targets for future research directed toward developing novel anti-*Campylobacter* treatments.

## *Campylobacter* Reservoirs and Sources of Infection

**FIGURE 1:**
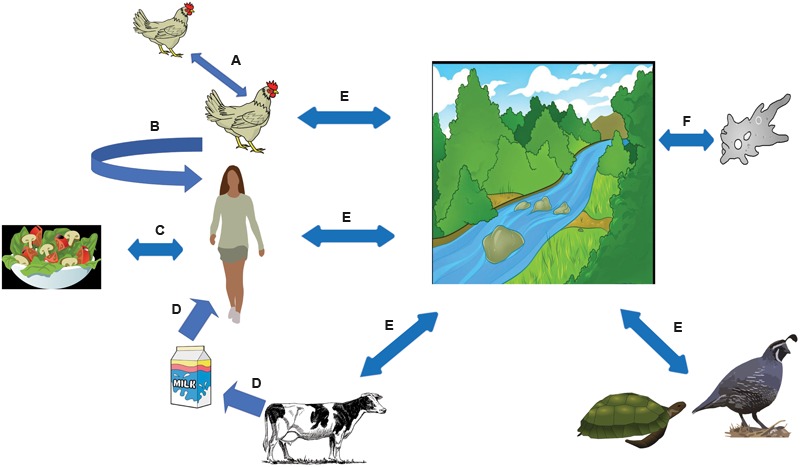
***Campylobacter* spp. modes of infection.** *Campylobacter* spp. reside in large numbers in the gastrointestinal tract of chickens, where the bacteria is spread throughout the flock via the fecal-oral route (A) (Young et al., 2007). In the developed world, *Campylobacter* is usually acquired by consuming under-cooked poultry (B). However, outbreaks have been associated with different types of fresh produce (C) (Kärenlampi and Hänninen, 2004) and dairy products (D) (El-Zamkan and Hameed, 2016). *Campylobacter* spp. is frequently found in surface water, usually from contamination from animal feces, and has been known to infect humans (E) (Mughini-Gras et al., 2016). It has also been postulated that *Campylobacter* may be able to infect amoebae, which may serve as a reservoir (F) (Axelsson-Olsson et al., 2005).

One factor that contributes to the widespread nature of human *Campylobacter* infections is the organism’s ubiquity in various domestic and wild animals (**Figure 1**). As mentioned above, avian species are the primary reservoir of *Campylobacter* spp., where they reside asymptomatically in large numbers within the lower gastrointestinal tracts of these animals (Johnson et al., 2015; Jonaidi-Jafari et al., 2016; Weis et al., 2016). As such, *Campylobacter* is commonly isolated from poultry, including chickens and turkeys, but also other domestic and wild avian species, such as crows, ducks, quail, and starlings (Jonaidi-Jafari et al., 2016; Weis et al., 2016). Even though the naturally high body temperature (40–42°C) (Johnston et al., 2016; Hamrita and Conway, 2017) of avian species provides an ideal environment for *Campylobacter* growth, the bacterium also commonly colonizes domestic livestock, including cattle, goats, pigs, and sheep (Manyi-Loh et al., 2016). For example, in beef and dairy cattle fecal samples in Finland, 31.1% of samples contained *Campylobacter* spp. (Hakkinen et al., 2007). Like many foodborne enteric pathogens, the presence of *Campylobacter* in so many animal species not only contributes to the prevalence of food-to-human transmission, but also environment-to-human transmission due to the abundance of agricultural contaminants in the environment.

Since *Campylobacter* can colonize such a broad range of animals, it is of interest whether some strains exhibit a predilection for certain hosts. Such observations would not only be useful in understanding the differences that may impart host preferences, but could also enable the epidemiological study of infection sources. In a genome-wide association study, Weis et al. (2016) compared *Campylobacter* strains isolated from humans, NHPs, chickens, cows, crows, goats, and sheep. The authors found that 17% of *Campylobacter* spp. isolated from crows were highly similar to those isolated from humans, primates, and sheep, indicating that multiple genotypes exist within individual bacterial species. With *C. jejuni*, it also elucidated host origin, providing evidence for host-species adaptation. Still, further investigation is needed to understand the genomes of naturally occurring *Campylobacter* strains from different environments and how they may provide evidence for host colonization mechanisms and zoonotic spread of the pathogen (Weis et al., 2016). As such, the study of these diverse strains and the insights they provide may yield promising targets for future research aimed at developing interventions that prevent transmission and persistence amongst animal reservoirs.

## *Campylobacter* Biofilms

Another trait of *Campylobacter* spp. that allows for environmental persistence is its ability to form biofilms on various abiotic surfaces, i.e., water distribution systems (Kalmokoff et al., 2006; Young et al., 2007; Maal-Bared et al., 2012; Bae et al., 2014; Duarte et al., 2016). Biofilms enable the microorganism to survive in environments it normally would not be able to, allowing it to acquire adequate nutrients while also providing protection from antimicrobials, including the disinfectants water sources are typically treated with (Simões et al., 2010). Thus, biofilms make it possible for *Campylobacter* to survive in water for up to 3 weeks and possibly longer (Lehtola et al., 2006). One mechanism in *Campylobacter* that is associated with biofilm formation is quorum sensing (QS). QS is a population-dependent cell-to-cell signaling mechanism involving the production and detection of extracellular signaling molecules (Elvers and Park, 2002). As QS communication has been linked to bacterial proliferation in foods and food spoilage, QS inhibition is a promising target to control *Campylobacter* and to ensure food safety (Nazzaro et al., 2013; Duarte et al., 2016). Since biofilm formation also increases the efficiency with which *Campylobacter* develops antibiotic resistance by horizontal gene transfer (discussed further below), the mechanisms responsible for biofilm formation are potential targets for future research aimed at mitigating the spread of genetic determinants responsible for resistance.

## Antibiotic Resistance Determinants in *Campylobacter*

As antibiotic resistance becomes increasingly prevalent in *Campylobacter*, the need for novel antimicrobial strategies to reduce *Campylobacter* in poultry and poultry products becomes more critical. This is primarily driven by the need to reduce the economic and human health burden incurred by antibiotic-resistant campylobacteriosis, (Duarte et al., 2016). A thorough comprehension of antibiotic resistance mechanisms in *Campylobacter* would aid in the development of novel anti-*Campylobacter* treatments, either by serving as targets themselves or by allowing for the development of strategies that circumvent resistance mechanisms.

*Campylobacter* can acquire antibiotic resistance by spontaneous mutations and horizontal gene transfer via natural transformation, transduction, and conjugation (Kumar et al., 2016). For example, the presence of conjugative plasmids containing *tetO*, have substantial roles in disseminating tetracycline resistance in *Campylobacter* (Pérez-Boto et al., 2014). Of the known antibiotic resistance determinants in *Campylobacter*, CmeABC is the best characterized (Martinez and Lin, 2006; Oh and Jeon, 2015). CmeABC is an energy-dependent multidrug efflux pump, and when the efflux pump inhibitor carbonyl cyanide *m*-chlorophenylhydrazone (CCCP) was added to *C. jejuni* 81–176 cultures, a rapid and substantial increase in cell-associated ciprofloxacin occurred (Lin et al., 2002). CmeABC consists of three protein components: the periplasmic fusion protein (CmeA), the inner membrane drug transporter (CmeB), and the OMP (CmeC) (Lin et al., 2002). As a result, an isogenic *cmeB* mutant of *C. jejuni* 21190 was found to be more susceptible to antibiotics (Lin et al., 2002). Antibiotic resistance in *Campylobacter* has also been shown to correspond to active site mutations in the DNA gyrase subunit A (*gyrA*) (Wieczorek and Osek, 2013; Kovač et al., 2015; Kumar et al., 2016).

In an attempt to directly target an antimicrobial resistance mechanism, it was previously shown that some phenolic compounds (i.e., gallic acid and taxifolin) significantly reduced the expression of the CmeABC efflux pump and that they could be used synergistically with antibiotics to inhibit *C. jejuni* by impacting both antimicrobial influx and efflux (Oh and Jeon, 2015). One promising use of phenolic compounds that has been proposed is the development of antimicrobial adjuvants, which would inhibit the function of resistant determinants, ultimately decreasing the ability of these strains to survive antibiotic treatment (Oh and Jeon, 2015). Since this approach would re-sensitize *Campylobacter* to antibiotics, it could augment the utility of existing antibiotics. Because of this, it has been suggested that phenolic compounds could be used as dietary supplements during antibiotic treatment of human campylobacteriosis (Wright, 2000; Pagès and Amaral, 2009; Oh and Jeon, 2015).

Since many *C. jejuni* strains are mutable and naturally competent, this species exhibits wide genetic diversity and variability, which increases the frequency of *Campylobacter* antibiotic resistance and virulence (Wilson et al., 2003; Young et al., 2007). Bae et al. (2014), reported that once *C. jejuni* develops resistance to antibiotics, those genetic determinants can be transferred in planktonic cultures, but are most efficiently transmitted in biofilms. C. *jejuni* exhibits donor restriction by an unknown mechanism as it takes up free DNA from *C. jejuni* more readily than it does from other bacterial strains (Wang and Taylor, 1990). The frequency of this transfer is also related to bacterial cell density since transformation efficiency correlates with increased bacterial numbers, presumably due to an increase of extracellular DNA in cultures (Wilson et al., 2003). *Campylobacter* also increases free DNA uptake under oxygen-limited conditions, like the gastrointestinal tract, which provides evidence for environmental regulation of horizontal gene transfer *in vivo* (Young et al., 2007). Due to the role these mechanisms play in the spread of antibiotic resistance, identifying interventions that inhibit natural transformation in *Campylobacter* are a goal of many researchers in the field.

## Anti-*Campylobacter* Compounds

Another area that has been pursued to reduce the prevalence of *Campylobacter* in agriculture, is the development of anti-*Campylobacter* compounds, such as small molecule inhibitors. Like similar work in other bacteria, these compounds can be directed against specific processes that are known to contribute to colonization or they can be developed as narrow-spectrum growth inhibitors. Whichever approach is taken; the goal is the same: the reduction of *Campylobacter* in agriculture using compounds that are not used in human medicine. Recently, Johnson et al. (2015) conducted a study to identify small molecule inhibitors of *Campylobacter* flagellar expression – a known colonization factor. Screening a library of approximately 147,000 small molecules, the authors identified compounds that modestly inhibited flagellar motility and several other compounds, termed ‘campynexins,’ that inhibited *Campylobacter* growth *in vitro* (Johnson et al., 2015). The campynexins exhibited robust growth inhibition with most inhibitory concentration 50s (IC50s) < 10 μM and, using *Helicobacter pylori*, were found to only inhibit members of the *Campylobacter* genus. Not surprisingly in these types of studies, the molecules of greatest interest are those that specifically exhibit activity toward *Campylobacter* – to minimize the effects on beneficial microbes in the gastrointestinal tract – and demonstrate efficacy *in vivo*.

According to Kumar et al. (2016), anti-*Campylobacter* small molecule inhibitors are considered bacteriostatic or bactericidal at a concentration of 200 μM. This study found 10 novel compounds with anti-*Campylobacter* activity, including molecules that induce intracellular clearance from human intestinal Caco-2 cells at concentrations as low as 25 μM (Kumar et al., 2016). A positive trait of these molecules is that they possessed low cytotoxicity to Caco-2 cells and no hemolytic activity against sheep red blood cells. The anti-*Campylobacter* molecules described in this study belong to five chemical classes that have been established as antimicrobial: aryl amines, piperazines, pyridiazinones, sulfonamides, and piperidines.

Treating *Campylobacter*-colonized chickens with these anti-*Campylobacter* molecules and evaluating the reduction in colonization is one area that should be investigated further. In the campynexin study, day-of-hatch chicks were used to evaluate the impact of the small molecules on gastrointestinal colonization (Johnson et al., 2015). While one of these compounds was found to significantly reduce *Campylobacter* loads *in vivo*, its lack of consistency indicates that further development from a medicinal chemistry perspective is needed. A potential extension of this work is the treatment of acute and persistent campylobacteriosis in immunocompromised patients that are infected with antibiotic resistant strains. Mice (Stahl et al., 2014), rats (Sung et al., 2013), and ferrets (Fox et al., 1987) have all been used as animal models for campylobacteriosis and should be considered as candidates for *in vivo* studies investigating the efficacy of anti-*Campylobacter* molecules at treating human infection.

Ideally, administration of anti-*Campylobacter* compounds will be as feed or water additives; however, there are several caveats with these approaches. Most obvious is that consumers are generally concerned with the use of synthetic additives in animal feed and their possible dissemination to the meat we consume. This has led researchers to pursue the development and use of natural additives in animal feed, rather than synthetic compounds (Verbeke et al., 2007; Brenes and Roura, 2010; Navarro et al., 2015). For example, phenolic compounds of plant origin have been shown to have anti-*Campylobacter* activity (Klanènik et al., 2012). In one study, compounds with the highest anti-*Campylobacter* activity were rosmarinic and carnosic acids. This study showed that inactivation of the efflux gene, *cmeB*, caused *Campylobacter* to be significantly more sensitive to the phenolic compounds, suggesting that transport of the compounds from the intracellular compartment is required for resistance.

Additionally, *Campylobacter* was shown to be sensitive to a variety of plant extracts including basil, campsicum, cinnamon bark, clove, garlic, laurel, lemon, lemon grass, lemon myrtle, mandarin, bitter and sweet orange, oregano, rosemary, sage, and thyme (Navarro et al., 2015). Several other plant-derived compounds, including anethole, carvacrol, cinnamaldehyde, citral, curcumin, eugenol, thymol, and vanillin have also been shown to have anti-*Campylobacter* activity, though the mechanism of toxicity is unknown (Navarro et al., 2015). In this study, oregano essential oil had the strongest anti-*Campylobacter* activity with a MIC of 0.0038% and formic acid was the most toxic organic acid with an MIC of 0.025%. Additionally, Lu et al. (2011) investigated the anti-*Campylobacter* activity of garlic and determined that organosulfur compounds were responsible at a greater level for antimicrobial activity than phenolic compounds. The antimicrobial activities of the garlic-derived organosulfur compounds increased as the number of sulfur atoms in the molecule increased. The greatest reduction in *Campylobacter* in this study was achieved with 25 μL garlic concentrate in broth incubated at 35°C. After 1 day, there were no viable *C. jejuni* cells detected in the medium containing garlic concentrate, compared to the control (medium with no garlic concentrate), which had 7.59 log_10_ CFU/mL *C. jejuni* (Lu et al., 2011).

In other work involving natural products that affect *Campylobacter* growth and viability, Šikić Pogačar et al. (2015) found that thyme ethanolic extract (TE), thyme post-hydrodistillation residue (TE-R), and OE reduced adhesion of *C. jejuni* to normal pig small intestinal epithelia-derived cells (PSI c1 cells) (Šikić Pogačar et al., 2015). Since adhesion to intestinal cells is necessary for colonization and disease, compounds that affect *Campylobacter* adherence may have as much disease-fighting potential as compounds that decrease the viability of *Campylobacter* (Pogačar et al., 2015).

The development of any of these natural products into effective anti-*Campylobacter* compounds could decrease the cost of feed formulations since it would eliminate the need of current antimicrobial feed additives (Navarro et al., 2015). As with the synthetic compounds proposed above, the utilization of natural additives as an anti-*Campylobacter* treatment, is an underexplored area and warrants more research to determine whether additives are transferred to meat products.

While some research has been conducted to identify and characterize anti-*Campylobacter* compounds, it is still lacking relative to the organism’s impact on agriculture and human health. Finding low-cost inhibitors will be essential in combating this increasingly antibiotic-resistant organism, either by mitigating colonization in commercial poultry or treating campylobacteriosis in humans. Regardless of the use, these compounds will need to be safe to both humans and livestock, and ideally would be narrow-spectrum in nature due to the increasing appreciation of the intestinal microbiota in the health of animals, including humans. Currently, neither synthetic nor natural compounds have been sufficiently shown to possess these traits, so much research into their efficacy remains to be performed.

## Probiotics as a Treatment for *Campylobacter* Colonization

Similar to the increasing appreciation of the intestinal microbiota in animal health, the use of probiotics as an effective means of preventing or reducing the incidence of *Campylobacter* infection in animal hosts in an antibiotic-free manner has garnered much interest (Fanelli et al., 2015; Kemmett, 2015). In addition to generally reducing the prevalence of *Campylobacter*, such a practice would, hypothetically, decrease the incidence of antibiotic-resistant strains since it would not require antimicrobials (Kemmett, 2015).

Previous studies that investigated probiotics as anti-*Campylobacter* treatments have appeared promising (Saint-Cyr et al., 2016). Several of these studies have focused on preventing *Campylobacter* colonization in broiler chickens at the primary production stage, typically by competitive exclusion of the pathogen by the probiotics (Bratz et al., 2015; Ştef, 2016; Thomrongsuwannakij et al., 2016). The mechanisms of competitive exclusion, includes the occupation of adhesions sites and receptors, secretion of antimicrobial substances, and competition for essential nutrients (Bratz et al., 2015). If a probiotic treatment were successful, such a practice could decrease the *Campylobacter* load in commercial poultry meat, making it safer for human consumption and reducing the incidence of campylobacteriosis (Fanelli et al., 2015). In addition to treating poultry, probiotics could potentially be used prophylactically for travel-related cases of campylobacteriosis or to treat persistent campylobacteriosis in regions of the world where it is endemic.

The probiotic genera that are most commonly evaluated for their ability to reduce *C. jejuni* colonization are *Lactobacillus, Bacillus*, and *Enterococcus*, as these are well characterized and commonly found in the intestines of animals (Arsi et al., 2015a,b; Thomrongsuwannakij et al., 2016). Researchers have also investigated the efficacy of *Bifidobacterium* spp. and *Saccharomyces cerevisiae* at inhibiting *C. jejuni* colonization and growth (Bratz et al., 2015; Fanelli et al., 2015).

A previous study postulated that *Lactobacillus acidophilus, Bacillus subtilis*, and *Enterococcus faecium* were the best probiotic candidates to combat *C. jejuni*. However, when broiler chickens were treated orally with each of these probiotic strains and later challenged with *C. jejuni*, there was no significant difference in *Campylobacter* numbers between treatment and control groups (Thomrongsuwannakij et al., 2016). In contrast, another group found that *Lactobacillus helveticus* strain R0052 reduced *C. jejuni* 81-176 and *C. jejuni* 11168 invasion of T84 cells by 41 and 35%, respectively (Wine et al., 2009). It was observed that *L. helveticus* adhered to the epithelial cells, suggesting that competitive exclusion may have contributed to the reduction in *C. jejuni* invasion (Wine et al., 2009). While this result was statistically significant, it was performed *in vitro* and such a modest reduction is unlikely to have much of an effect on product safety. In a similar study, 117 bacterial species found in the ceca of broiler chickens, were screened, and three bacterial species were determined to significantly decrease *Campylobacter* colonization of chickens (Arsi et al., 2015a).

Another study reported that multiple *Lactobacillus* strains inhibited the growth of *C. jejuni in vitro* due to organic acid production by these microorganisms (Bratz et al., 2015). *Lactobacillus* spp. lower pH to create a more hospitable environment for themselves, an effect that is increased when multiple strains are present (Wang et al., 2014; Kemmett, 2015; Wooten et al., 2016). Unfortunately, using probiotics to eliminate *Campylobacter* solely by lowering pH may not be efficacious *in vivo* since the lower gastrointestinal tract is highly buffered by bicarbonate present in pancreatic juices.

An additional study screened 116 bacteria and reported six strains (*Bacillus* spp.) that reduced *C. jejuni* counts by at least 1–2 log_10_ *in vivo* (Arsi et al., 2015b). These results suggest intracloacal administration of probiotics to broiler chickens is effective and would eliminate the need for encapsulation of the probiotic (Arsi et al., 2015b). Unfortunately, such administration is likely prohibitive from a labor perspective since intracloacal administration of probiotics to large flocks would require a tremendous amount of effort from producers.

Prebiotics, non-digestible food ingredients that promote beneficial bacterial growth in the gut, have also been used to reduce the prevalence of *Campylobacter* in the broiler chicken gastrointestinal tract. Although they showed no significant impact on their own, prebiotics did significantly decrease the amount of *Campylobacter* when used in combination with three probiotic species (Arsi et al., 2015a). Similar studies supported these results where *Campylobacter* loads were reduced in the presence of a combination of prebiotics and probiotics (Peng et al., 2015; Gracia et al., 2016; Guyard-Nicodeme et al., 2016).

Similar to the studies above using bacterial probiotics, *S. cerevisiae* was also found to have an inhibitory effect on *Campylobacter*. When administered to broiler chickens as a supplement, *S. cerevisiae* was shown to significantly decrease the amount of both *Campylobacter* and *Salmonella* in the cecum, feces, breast skin, and neck skin. It was determined this occurred because *S. cerevisiae* promoted *Lactobacillus* growth, which competed with *Campylobacter* and *Salmonella* for nutrients and attachment sites in the intestines (Fanelli et al., 2015). Taken with the above studies, this treatment is likely not feasible since the direct administration of several *Lactobacillus* species was unable to induce appreciable reductions in *Campylobacter* colonization. Also, the promotion of *Lactobacillus* growth in the presence of *S. cerevisiae* appears contradictory since their abundance in multiple environments is often inversely related. Thus, further studies into the efficacy of *S. cerevisiae-*induced inhibition of *Campylobacter* should be performed.

In addition to potentially reducing *Campylobacter* numbers, probiotics can provide several other benefits to their hosts. For example, when either two or four *Lactobacillus* strains were added to feed, chickens displayed an increase in metabolic rate, nutrient transport capacity, protein production, and adaptability and response to external factors. These effects were most pronounced in chickens administered four *Lactobacillus* strains from the day they hatched (Ştef, 2016).

Based on the data above, it appears that the use of probiotics is occasionally effective at reducing *Campylobacter* colonization in chickens, but the methodology and significance of those reductions is somewhat questionable. Some of this confusion is due to large discrepancies that have been observed in these studies (Meunier et al., 2016a). Several factors could explain this variation, including the use of different chicken lines, since their sensitivity to *Campylobacter* or probiotic treatments may vary (Humphrey et al., 2014). Also, differences in *Campylobacter* strains and doses, as well as differences in administration routes and timing, could lead to these observed discrepancies (Meunier et al., 2016a). Still, Saint-Cyr et al. (2016) proposed that probiotic studies should combine various *in vitro* and *in vivo* methods to better account for host complexity, animal feed, and the microbiota. For example, the *in vitro* models that have been used to investigate anti-*Campylobacter* activities of probiotics have been based on human cervical or intestinal cell lines. Not surprisingly, the use of avian cell lines may provide a better model if the goal is to identify probiotics that can be used in chickens (Saint-Cyr et al., 2016). Additionally, it may be worthwhile to investigate different and varied bacterial strains to develop an effective anti-*Campylobacter* probiotic treatment (Saint-Cyr et al., 2016). Lastly, searching for bacterial species that can drastically reduce colonization of *Campylobacter* in an environment where both already reside may not be the most efficient approach. Instead, researchers may identify more inhibitory probiotics looking elsewhere, with the caveat that those organisms would need to be proficient at colonizing poultry and cannot exert a negative impact on the health or production of the bird. As such, there is likely much more work needed before an effective probiotic is available.

A potential future direction of these probiotic studies would be to determine if the probiotic benefits and *Campylobacter*-inhibiting capabilities would be similar in an animal model of human campylobacteriosis. It has been difficult to conduct this type of research because an animal model that mimics human campylobacteriosis is not frequently used (Mohan, 2015). However, as mentioned above, there are several animals [i.e., mice (Stahl et al., 2014), rats (Sung et al., 2013), and ferrets (Fox et al., 1987)], that have shown potential for this application, but further work needs to be performed to determine their effectiveness.

## *Campylobacter* Bacteriophage as a Treatment

Bacteriophages have garnered considerable interest as potential treatments to reduce *Campylobacter* colonization in commercial poultry. Bacteriophages are viral predators of bacteria that are ubiquitous in the environment and often exhibit exquisite specificity against their host bacterial species. Bacteriophages could potentially be used without impacting the normal microbiota of the host and may be suitable for reducing *C. jejuni* colonization at the farm level, thus decreasing transmission to the food chain. These attributes make bacteriophages an attractive anti-*Campylobacter* treatment (El-Shibiny et al., 2009). Thus, the use of bacteriophage as an intervention strategy has been pursued by several research groups (Atterbury et al., 2005; Brüssow et al., 2007; Connerton et al., 2008; El-Shibiny et al., 2009).

Bacteriophages that are effective against *Campylobacter* have been isolated from multiple sources, including sewage, pig manure, poultry carcasses, and broiler chickens (Grajewski et al., 1985; Salama et al., 1989; Atterbury et al., 2003; El-Shibiny et al., 2005, 2009; Carrillo et al., 2007; Hansen et al., 2007). These bacteriophages, including those identified by Atterbury et al. (2003), represented a spectrum of different lytic classes that can be readily imaged by electron microscopy (El-Shibiny et al., 2009).

Atterbury et al. (2005) determined that *C. jejuni* counts in broiler chickens was significantly lower when bacteriophages were present than when they were absent; means of 5.1 log_10_ CFU/g in chickens with bacteriophage and 6.9 log_10_ CFU/g in chickens without bacteriophage. Connerton et al. (2008) reported reductions of 2–5 log_10_ CFU of *Campylobacter* per gram of chicken cecal contents following treatment with bacteriophage. El-Shibiny et al. (2009) reported that a 7 log_10_ PFU dose of the *Campylobacter*-specific bacteriophage, CP220, led to a 2 log_10_ CFU/g decline in *Campylobacter* counts 48 h post-phage inoculation. Since these reductions are in the range of the 2 log_10_ reductions mentioned earlier, which are hypothesized to result in a 30-fold reduction in human infections, bacteriophages could potentially have a significant impact on human health. Unfortunately, one area these studies are lacking in is the analysis of the chicken microbiota in response to treatment. As with all the strategies above, the goal of these interventions would be to reduce *Campylobacter* loads specifically while sparing the beneficial inhabitants of the microbiota.

Utilizing bacteriophage to reduce *Campylobacter* loads in chickens has shown potential at the lab scale; however, there is still work to be done before it can become a feasible treatment at the farm level. The bacteriophage titers necessary to cause a significant reduction in *Campylobacter*, needs to be minimized. For example, the 7 log_10_ PFU that was needed for a 2 log_10_ reduction of *C. jejuni* (mentioned above) or the 9 log_10_ PFU that was needed for a similar reduction of *C. coli* (El-Shibiny et al., 2009), indicates that it would not be feasible to treat every chicken on a large farm with bacteriophage doses of these sizes.

Another barrier to developing a successful *Campylobacter* bacteriophage treatment is that phage, like most predators, seldom eliminate their prey in nature. Rather, the populations of bacteriophage and target bacteria rise and fall in a cyclic manner (Wagenaar et al., 2005; El-Shibiny et al., 2009; Grant et al., 2016). Also, *Campylobacter* may use genomic instability to avoid predation from phage; however, bacteriophages constantly evolve to circumvent host barriers to infection (Carrillo et al., 2005; El-Shibiny et al., 2009). Also, to develop a successful *Campylobacter* bacteriophage therapy, the phage must also be able to tolerate gastric pH (El-Shibiny et al., 2009). All of the potential barriers to a successful therapy described above must be addressed before *Campylobacter* bacteriophage can become feasible at the farm level. It has been postulated that this success will also be based on inoculum volume, inoculation timing, bacteriophage absorption rate, and burst size (Connerton et al., 2011).

While several studies have shown bacteriophage treatment can reduce *Campylobacter* loads in commercial chickens, to the best of our knowledge, no studies have evaluated the capability of bacteriophage to treat *Campylobacter* colonization in humans. Bacteriophage therapy was used widely throughout the 20th century in Eastern Europe and the former Soviet Union; however, it has not yet been investigated by rigorous scientific standards (Pelfrene et al., 2016). With the increasing incidence of antibiotic-resistant bacteria, including *Campylobacter*, it has begun to be re-evaluated as a potential therapeutic for use in human disease. As such, it would be interesting to evaluate the efficacy of bacteriophage at treating human infections. As mentioned above, several animal models exist [i.e., mice (Stahl et al., 2014), rats (Sung et al., 2013), and ferrets (Fox et al., 1987)], that can help determine the effectiveness of bacteriophage, as well as fermentor systems that have previously been used to simulate gut function (Sumeri et al., 2008; Neuman et al., 2014; Kettle et al., 2015).

Due to the relatively low and transient *Campylobacter* numbers that occur during human infection, it is anticipated that the number of bacteriophage required to reduce colonization during infection, may be lower than those needed to treat the robust and stable population that occurs in chickens. Additionally, the usual specificity of bacteriophage for their host means that treatment may spare beneficial members of the microbiota. This hypothesis is supported by the observation that several types of bacteriophages that exhibit effects toward *Campylobacter* have been isolated from sources humans are readily exposed to (waterways, livestock, etc.) without any known effects on human gastrointestinal health.

Like any other intervention against *Campylobacter*, there is a concern that over time the bacteria will develop resistance to the bacteriophage. Fortunately, in a previous study, the incidence of bacteriophage resistance developing in *C. jejuni* colonized chickens was 2% and the resistant strains remained a minor component of the population (El-Shibiny et al., 2009). Still, various types of bacteriophage could be used in combination to maintain *Campylobacter*-free chickens. This may be necessary even in the absence of resistance because, to date, no bacteriophage has exhibited pan-effectiveness against every *Campylobacter* strain examined. Additionally, microbial resistance to bacteriophage has been correlated with reduced virulence *in vivo*, indicating that even if a population becomes resistant, it could still benefit human health (Smith et al., 1987; Connerton et al., 2004; Carrillo et al., 2005; El-Shibiny et al., 2009).

At this time, the United States Food and Drug Administration has not approved the pre-harvest use of bacteriophage as an antimicrobial agent. However, a substantial amount of research is currently being conducted globally, which could lead to an accepted treatment (Grant et al., 2016).

## *Campylobacter* Vaccines for Poultry and Humans

Like the interventions proposed above, vaccination of poultry against *Campylobacter* could eliminate the microorganism from birds and reduce the incidence of human campylobacteriosis in the developed world (Avci, 2016). Not only would this reduce the occurrence of chicken-to-human *Campylobacter* transmission, but would also reduce the need for expensive post-harvest treatments (De Zoete et al., 2007; Saxena et al., 2013). At the farm level, *Campylobacter* has no direct influence on chicken health, productivity, or farmer income (Shane, 2000), thus the farmer would have little incentive to invest resources to reduce the incidence of *Campylobacter* on the farm. However, the cost of campylobacteriosis to public health systems and the loss of labor productivity is substantial, therefore the main rationale for developing a *Campylobacter* vaccine would be to reduce potential human health risks, enhance food safety, and decrease the high costs associated with the disease. For the reasons described above, the need for a *Campylobacter* vaccine may not be driven by the market itself, but will likely require intervention by government agencies (Lund and Jensen, 2016).

Despite the substantial amount of research directed toward vaccine development, currently there is no vaccine on the market to reduce *Campylobacter* loads in the gastrointestinal tract of chickens (Meunier et al., 2016b). A summary of the antigens used as candidates for *Campylobacter* vaccines are shown in **Table 1**. A vaccine has recently been patented that is comprised of a bacterium engineered to produce at least one *Campylobacter* derived *N*-glycan, and at least one physiologically acceptable diluent, excipient, adjuvant, or carrier (Szymanski and Nothaft, 2016). In this patent, chickens exposed to a ToxC-GT glycoconjugate had a significant reduction of *Campylobacter* in the cecal contents of challenged chickens. According to the developers, this vaccine composition can be formulated for addition to livestock feed and for administration to poultry.

**Table 1:** Antigens used as candidates for *Campylobacter* vaccines.

Antigen	Reference
ToxC-GT glycoconjugate	Szymanski and Nothaft, 2016
CjaA	Wyszyńska et al., 2004; Buckley et al., 2010
CadF, FlpA, CmeC, and Dsp	Theoret et al., 2012; Neal-McKinney et al., 2014
Total outer membrane proteins	Annamalai et al., 2013
Fusion proteins	Neal-McKinney et al., 2014
Extracytoplasmic proteins	Zeng et al., 2010; Layton et al., 2011; Clark et al., 2012; Neal-McKinney et al., 2014; Kobierecka et al., 2016
*Campylobacter* flagellin	Khoury and Meinersmann, 1995; Widders et al., 1998; Huang et al., 2010; Tribble et al., 2008; Meunier et al., 2016b; Riddle and Guerry, 2016
Whole cell vaccine (*C. jejuni* 81–176)	Tribble et al., 2008
Protein subunit vaccine	Maue et al., 2014
*Campylobacter* capsule polysaccharide	Schumack et al., 2016

Other antigens that have been investigated as subunit vaccines for chickens are the periplasmic protein, CjaA, (Buckley et al., 2010) and the adherence and colonization proteins, CadF, FlpA, CmeC, and Dsp (Theoret et al., 2012; Neal-McKinney et al., 2014). Total OMPs (Annamalai et al., 2013) and fusion proteins (Neal-McKinney et al., 2014) have also been evaluated (Meunier et al., 2016b).

A previous study showed that chicken immunization with an avirulent *Salmonella* strain expressing *Campylobacter* CjaA substantially reduced the ability of *C. jejuni* to colonize chicken ceca. The authors reported an approximately 6 log_10_ CFU/g reduction in cecal contents (Wyszyńska et al., 2004). A more recent study reported the live-attenuated *Salmonella* vaccine expressing *Campylobacter* CjaA led to a significant, but far less prominent, reduction of 1.4 log_10_ CFU/g *C. jejuni* in chicken cecal contents (Buckley et al., 2010). Similarly, another group evaluated the efficacy of a recombinant attenuated *Salmonella enterica* strain synthesizing the Dsp protein and observed a 2.5 log_10_ reduction of *C. jejuni* in chicks after a homologous challenge (Theoret et al., 2012). Neal-McKinney et al. (2014) evaluated several recombinant *C. jejuni* peptides and a fusion protein as chicken vaccines and determined that the greatest reduction in *C. jejuni* colonization was in chickens injected with a recombinant FlaA or FlpA peptide, or a CadF-FlaA-FlpA fusion protein. These vaccinations all resulted in a greater than 2 log_10_ reduction in *C. jejuni* colonization. Advanced delivery systems have also been evaluated; biodegradable and biocompatible poly (lactide-co-glycolide) NP encapsulated OMPs of *C. jejuni* were used to vaccinate chickens (Annamalai et al., 2013). In this study, *C. jejuni* colonization of the chicken cloaca and ceca were below the limit of detection in the vaccinated groups following 7-days post-challenge.

Another protein that has been investigated as a potentially effective immunogen is *Campylobacter* flagellin, which is the immunodominant *Campylobacter* antigen (Meunier et al., 2016b). Studies have shown induction of an immune response toward *Campylobacter* flagellin, but this was not correlated with a decrease in colonization of the chicken gut (Khoury and Meinersmann, 1995; Widders et al., 1998; Huang et al., 2010; Meunier et al., 2016b).

Less targeted approaches using numerous conserved extracytoplasmic proteins have also been evaluated for *Campylobacter* vaccine development (Wyszyńska et al., 2004; Buckley et al., 2010; Zeng et al., 2010; Layton et al., 2011; Clark et al., 2012; Neal-McKinney et al., 2014; Kobierecka et al., 2016). In these studies, the median reduction of *C. jejuni* in chicken cecal contents ranged from 6 log_10_ (Wyszyńska et al., 2004) to less than 1 log_10_ (Buckley et al., 2010). In these two studies, vaccines were administered at comparable doses on identical days post-hatch. However, Wyszyńska et al. (2004) did not report the course of intestinal colonization, systemic translocation of the vaccine strain, or whether the vaccine strain was present at the point of challenge (Buckley et al., 2010). Buckley et al. (2010) suggested that the line and immune status of the chicken could have attributed to the substantially different results. Regardless, there is a consensus that immunodominant, surface-located proteins are more potent antigens since they are more accessible for inducing antibody production. For this reason, as evidenced in the above work, most antigens vetted for *Campylobacter* vaccine development have been extracellular in nature (Kobierecka et al., 2016).

In addition to development of poultry vaccines, there has been a considerable amount of research toward the development of a *Campylobacter* vaccine for humans, which would be primarily marketed toward travelers and the military. There have been several candidates that have advanced to human testing; however, none of these candidates has been able to confer sufficient protection to date (Maue et al., 2014).

One category of human *Campylobacter* vaccine that has generated considerable interest is subunit vaccines. Generally, *C. jejuni* strains produce lipo-oligosaccharides (LOS) that contain *N*-acetyl neuraminic acid moieties that are molecular mimics of human gangliosides. Unfortunately, antibodies directed against these mimics may cross-react with human peripheral nerves, which is the pathogenic basis of GBS. Thus, whole cell oral vaccines that are at times logical for developing protection against other enteric pathogens, are not the preferred approach for vaccine development against *Campylobacter* (Riddle and Guerry, 2016). Regardless, a whole cell vaccine was developed, but it was unsuccessful in a phase 2b challenge with *C. jejuni* 81–176 (Tribble et al., 2008).

Several subunit vaccines have been pursued. A flagellin subunit protein vaccine was only slightly immunogenic in phase 1 testing (Tribble et al., 2008). ACE Biosciences developed a protein subunit vaccine that was determined to be non-effective in phase 2b trials (Maue et al., 2014). Another recent study showed that a recombinant non-glycosylated *C. jejuni* flagellin was poorly immunogenic in Phase I trials and would likely not be effective (Riddle and Guerry, 2016). Schumack et al. developed a conjugate vaccine against the *Campylobacter* capsule polysaccharide (CPS) that conferred 100% protection against diarrhea from a homologous *C. jejuni* strain in a NHP model (Schumack et al., 2016). While promising, the mechanism of protection from this vaccine remains unknown.

Much effort has been leveraged toward vaccine development, since it is generally considered the most effective strategy to prevent diseases caused by viral and bacterial pathogens (Kobierecka et al., 2016). While there are obvious advantages to developing an effective *Campylobacter* vaccine, there are several hurdles that must be overcome. Unfortunately, vaccination is expensive due to the development and manufacturing processes. These stages are costly, complex, and lengthy, and are coupled with considerable economic and technological uncertainty (Lund and Jensen, 2016). Other disadvantages associated with vaccines is that storage is costly and time limited, and adjustment of the productive capacity is slow, expensive, and overseen by regulation. Vaccine production is characterized by economies of scale and is subject to large-scale errors (i.e., batch failures). As such, only a small fraction of all vaccine candidates reach the market (Jensen et al., 2014). In addition to these more general challenges, *Campylobacter* vaccine development is currently hindered by an incomplete comprehension of their protective epitopes, antigenic diversity, pathogenesis, and their association with post-infectious syndromes such as irritable bowel syndrome, reactive arthritis, and GBS (Riddle and Guerry, 2016).

## Anti-*Campylobacter* Bacteriocins

Bacteriocins are another potential treatment option that has been pursued to reduce the incidence of *Campylobacter* colonization in chickens. These proteinaceous compounds, synthesized by other bacteria, target and reduce the viability of closely related bacteria (Quereda et al., 2016). Typically, anti-*Campylobacter* bacteriocins are microencapsulated and administered to poultry through chicken feed. For an earlier detailed review of bacteriocins and their potential to inhibit *Campylobacter* in poultry, we suggest the article published by Svetoch and Stern (2010).

Prior to determining the efficacy of the bacteriocins *in vivo*, the proteins can be purified and characterized *in vitro* using inhibition zone diameter as the basis for selecting favorable anti-*Campylobacter* bacteriocins. This was done to describe four bacteriocins from different strains of *Paenibacillus polymyxa* and *Bacillus circulans* NRRL B-30644 (Svetoch et al., 2005). More recently, Messaoudi et al. (2012) used a purified bacteriocin from *L. salivarius* SMXD51 to decrease *C. jejuni* viability *in vitro* by 2-log_10_ compared to an untreated control.

Following identification, purified bacteriocins can be administered to colonized birds. Stern et al. (2006) purified bacteriocin OR-7 from *Lactobacillus salivarus* NRRL B-30514 and treated chickens colonized with *C. jejuni*. Treatment with this bacteriocin reduced *C. jejuni* colonization of chickens by at least 6 log_10_ compared to untreated groups (Stern et al., 2006). In another study by Stern et al. (2005), a class IIa bacteriocin secreted by *Paenibacillus polymyxa* NRRL B-30509 was purified and incorporated into chicken feed. Consistently, significant reductions in colonization by *C. jejuni* were observed and in one part of the study, no viable *C. jejuni* were detected in these chickens. This was in contrast to untreated birds that were colonized at a mean of 7.2 log_10_ CFU/g feces (Stern et al., 2005). Similarly, another group utilized bacteriocin B602, secreted by *P. polymyxa* NRRL B-30509, and OR7, secreted by *L. salivarius* NRRL B-35014, to reduce *C. coli* colonization in turkey poults. In each of three separate trials, *C. coli* concentrations were below the level of detection in the ceca and duodenum (Cole et al., 2006).

To further develop these proteins as a treatment, subsequent studies determined whether cell-free supernatants or co-infection with bacteriocin producing strains were sufficient to eliminate *Campylobacter* colonization. In one study, the supernatants of *L. salivarius* SMXD51, *L. salivarius* MMS122, and *L. salivarius* MMS151 were shown via the formation of inhibition zones on *C. jejuni* and *C. coli* lawns to possess anti-*Campylobacter* compounds (Messaoudi et al., 2011). The authors concluded that bacteriocins were the cause of this inhibition as adding a proteinase led to a lack of inhibition from the supernatant. Presumably, using supernatant rather than purified bacteriocins would be preferred from an industrial standpoint, as purifying bacteriocins would add to the labor and cost of the final product (Messaoudi et al., 2011). Unfortunately, when viable *L. salivarius* NRRL B-30514 and *P. polymyxa* NRRL B-30509 were used as antagonists against *Campylobacter*, there was no inhibitory effect observed; this contrasted with the 6 log_10_ reduction that was observed in chickens using bacteriocins purified from these strains (Stern et al., 2008). Based on this and the Messaoudi et al. (2012) study, supernatants from bacterial cultures may be a viable anti-*Campylobacter* treatment. However, using the bacterial strains directly as a probiotic does not seem efficacious for eliminating *Campylobacter* colonization.

As with any anti*-Campylobacter* treatment, there is the possibility strains will develop resistance to the compound. A screen for *C. jejuni* and *C. coli* isolates that developed resistance to the bacteriocins OR-7 and E-760 identified a *C. coli* strain that was significantly resistant. Analysis of this strain revealed that the multidrug efflux pump CmeABC contributed to both acquired and intrinsic resistance of the strain to the bacteriocins (Van Hoang et al., 2011b). A companion study showed that the low level of bacteriocin resistance developed by *C. jejuni* strains was not stable in the absence of selective pressure from the bacteriocins. This suggests that, while bacteriocin resistance may need to be addressed when its use becomes more widespread, the impact will likely remain transient (Van Hoang et al., 2011a).

Lastly, as with many of the above treatments, the effect of the bacteriocins on the poultry gastrointestinal microbiota is currently unknown. Fortunately, this strategy will likely be employed shortly before harvest, so concerns about the bacteriocins affecting the microbiota and influencing production efficiency are likely unfounded. Still, it would bolster the attractiveness of this approach if the specificity of the bacteriocins were known. It also needs to be determined whether the bacteriocins contaminate meat products following harvest. If this is found to occur, their stability and their effect on the human gastrointestinal environment may need to be investigated to maintain interest in this approach.

## Conclusion

As the incidence of antibiotic resistant *Campylobacter* strains is increasing, the need for the development of novel non-antibiotic anti-*Campylobacter* treatments is becoming more critical. As such, much research is being conducted to develop treatments that either reduce *Campylobacter* colonization in chickens or eliminate acute infections in humans. Treatment strategies that are currently under development include anti-*Campylobacter* compounds, probiotics, *Campylobacter*-specific bacteriophage, chicken and human *Campylobacter* vaccines, and anti*-Campylobacter* bacteriocins. While several of these approaches have proven promising, it is apparent that further research is required to develop these into truly efficacious treatments. Regardless, it is encouraging that so many avenues have been, and are currently being, pursued by several talented research groups.

Further, it is necessary that the mechanisms of colonization and pathogenesis for both animals and humans is well understood, since it is likely to lead to the identification of more targets that can be used for the development of different interventions, like those mentioned above. This research need is particularly urgent for under-researched areas, like the impact of persistent *Campylobacter* colonization on the health of young children in the developing world. Hopefully, it is apparent here that the global health burden caused by *Campylobacter* is substantial and that these concerns are compounded by the burgeoning rates of antibiotic resistance observed in this microorganism. As such, the need to develop novel treatment strategies and conduct further research into colonization and disease mechanisms is essential in mitigating the negative effects of *Campylobacter* on global human health.

## Author Contributions

TJ, JS, and JJ conceived, designed, wrote, and edited this manuscript for critical content. All authors approve this manuscript for content integrity and accuracy.

## Conflict of Interest Statement

The authors declare that the research was conducted in the absence of any commercial or financial relationships that could be construed as a potential conflict of interest.

## Abbreviations

CCCP: carbonyl cyanide *m*-chlorophenylhydrazone
CPS: capsule polysaccharide
EO: essential oil
GBS: Guillain–Barré syndrome
IC50: inhibitory concentration 50
LOS: lipo-oligosaccharides
MIC: minimum inhibitory concentration
NHP: non-human primate
NP: nanoparticle
OE: olive leaf extract
OMP: outer membrane protein
QS: quorum sensing
RND: resistance-nodulation-cell division
TE: thyme ethanolic extract
TE-R: thyme hydrodistillation residue
